# Oceanic Life at Great Depths

**Published:** 1869-03

**Authors:** 


Oceanic Life at Great Depths.—At a meeting of the Royal Microscopical Society on Wednesday night, the 9th instant, Dr. Carpenter anticipated his paper already presented to the Royal Society by offering some remarks on, and exhibiting specimens illustrative of, his recent deep-sea inquiries. It will be in the memory of our readers that in the autumn Dr.

Carpenter and Professor Wyville Thompson, of Queen's College, Belfast, went out, under government auspices, to conduct certain biological and physical investigations into the characters of the fauna and the temperature of the water at great depths. The expedition was carried out with the greatest success, and with the most remarkable results. The researches of Dr. Wallich and other able zoölogists had determined that the dictum of Forbes respecting the depth at which animal life existed was unfounded, and that animals existed at a depth of several hundred fathoms in the ocean. Dr. Carpenter now confirms this fact, and extends it in many important directions. In the very interesting sketch of his excursion given by Dr. Carpenter it was shown that even at depths so great as 650 fathoms in a line south-west (?) of the Faroe Islands the dredge brought up not only Globigerinæ and similar low forms of animal life, but representatives of all the great invertebrate types. Many of the specimens of Protozoa captured at this depth were exhibited by Dr. Carpenter, and some of them were certainly extraordinary samples of classes whose members are generally extremely minute. Indeed, these discoveries of Dr. Carpenter open up to us quite a new phase of animal life, and reveal a vast field for physiological speculation and inquiry. Of the specimens exhibited under the microscope we noted the following: Cristelaria, Textularia, Cornuspira, Trochamina, Lituola, Rhabdammina and Astrorhiza limicola. The last especially is a most remarkable creature, and one likely, we should think, to afford zoölogists many difficult problems for solution. In reference to the results of the physical discoveries, one of the most singular and noteworthy is that of temperature. Several carefully conducted soundings have convinced Dr. Carpenter and his colleague that whilst the surface water has an almost invariable temperature of 52°, the heat at great depth varies exceedingly. Thus at one point in their voyage they found, at a depth of 500 fathoms, the temperature was 32°—a fact which is explained by the supposition of a cold Arctic stream flowing from the north-east, and apparently coming between the fork of the Gulf Stream. Another interesting fact established by these inquiries is, that even at a temperature in the ocean almost that of our freezing-point there are an abundance and variety of animal forms which could not have been predicated. Dr. Carpenter's researches will soon be laid before the public *in extenso*, but in the meantime we could not resist bringing the above extraordinary and unexpected results under the notice of our readers.—*Medical Times and Gazette*, *Dec. 12*, *1868*, and *Med. News*.

